# Purple grape juice improves performance of recreational runners, but the effect is genotype dependent: a double blind, randomized, controlled trial

**DOI:** 10.1186/s12263-022-00710-1

**Published:** 2022-06-02

**Authors:** Bruno Rafael Virginio de Sousa, Lydiane de Lima Tavares Toscano, Eder Jackson Bezerra de Almeida Filho, Klécia Farias Sena, Matheus Silveira Costa, Rebeka Correia de Souza Cunha, Jullyana de Souza Siqueira Quintans, Luana Heimfarth, Aline Telles Biasoto Marques, Darcilene Fiuza da Silva, Luis Felipe Castelli Correia de Campos, Darlene Camati Persuhn, Alexandre Sérgio Silva

**Affiliations:** 1 Programa de Pós-graduação em Ciências da Nutrição and Laboratório de Estudos do Treinamento Físico Aplicado ao Desempenho e a Saúde, Departamento de Educação Física, Universidade Federal da Paraíba (UFPB), João Pessoa, Paraíba Brasil; 2 Laboratório de Estudos do Treinamento Físico Aplicado ao Desempenho e a Saúde, Departamento de Educação Física, Universidade Federal da Paraíba (UFPB), João Pessoa, Paraíba Brasil 58059-900; 3 Programa de Pós-graduação em Ciência e Tecnologia de Alimentos, Universidade Federal da Paraíba (UFPB), João Pessoa, Paraíba Brasil; 4 Laboratório de Neurociências e Ensaios Farmacológicos, Universidade Federal de Sergipe (UFS), Aracajú, Sergipe Brasil; 5 Empresa Brasileira de Pesquisa Agropecuária, Embrapa Semiárido, Petrolina, Pernambuco Brasil; 6 Faculdade de Farmácia, Universidade Federal da Bahia, Salvador, Bahia Brasil; 7 Departamento de Ciências da Educação, Universidade de Bío-Bío, Concepción, Chile; 8 Departamento de Biologia Molecular, Universidade Federal da Paraíba (UFPB), João Pessoa, Paraíba Brasil

**Keywords:** Antioxidant, Ergogenic food, Polymorphisms, Nutrigenetic

## Abstract

**Background:**

We examined the influence of superoxide dismutase 3 (SOD3) Arg213Gly and Peroxisome Proliferator-Activated α-Receptor (PPARα) 7G/C polymorphisms to a single dose of purple grape juice supplementation on time-to-exhaustion running test, redox balance and muscle damage in recreational runners.

**Methods:**

Forty-seven male recreational runners performed a running test until exhaustion after supplementation with grape juice or a control drink. Serum total antioxidant capacity (TAC), malondialdehyde (MDA), plasma nitrite (NO), creatine kinase (CK) and lactate dehydrogenase (LDH) were measured pre and post exercise. Also, polymorphisms were analyzed in DNA extracted from the oral mucosa.

**Results:**

Grape juice improved the time-to-exhaustion. When analyzed by genotype, the recreational runners with GG+CG genotypes of the SOD3 gene had greater time-to-exhaustion than the CC genotype, but was no different for the PAPRα gene. A slight difference was noted in TAC, since the CC genotype of the SOD3 gene showed higher TAC values in the post-exercise compared to the baseline and with pre-exercise, but these values did not increase compared to the CG+GG group, respectively. The SOD3 and PPARα genes were similar at all times for the other biochemical variables.

**Conclusion:**

The ergogenic effect of grape juice was genotype-dependent for SOD3 Arg213Gly. However, biochemical redox balance markers did not explain this difference.

## Background

Purple grapes and derivatives present high antioxidant and anti-inflammatory properties [1, 2]. These characteristics are attributed to their rich composition in flavonoids. These include flavanols, (catechin, epicatechin and proanthocyanidins), flavonols (quercetin, kaempferol, myricetin, laricitrin, isorhamnetin and syringetin) and anthocyanins (malvidin, cyanidin, peonidin, delphinidin, pelargonidin and petunidin) [3], as well as non-flavonoid compounds, such as phenolic acid and resveratrol [4]. This antioxidant action was previously demonstrated in studies in the field of exercise training in which purple grape seed extract [5] and purple grape bagasse extract [6, 7] reduced oxidative stress markers in animal model.

In humans, recreational runners supplemented with purple grape juice (10 mL/kg/day for 28 days) increased their time-to-exhaustion running test time by 15%, accompanied by an increase in total antioxidant capacity and reduction in inflammatory activity, even without any protection against muscle damage induced by the exercise session [8]. This ergogenic effect of the purple grape was recently confirmed in another study that employed a single dose (10 mL/kg). It increased the time-to-exhaustion in runners (18.7%) to an even greater extent, accompanied by increased total antioxidant capacity [9].

Although promising, these outcomes displayed high variability in response to supplementation, in which over 28 days [8] 67% were responsive to grape juice improved performance within a range of 3% to 73%, while 33% were not responsive to grape juice. In the study in which a single dose was administered [9], improvement among 70% who showed responsiveness to juice and improved performance was between 1% and 74%, and 30% were not responsive to grape juice.

In an attempt to explain this variability found in response to purple grape juice supplementation, genetic polymorphisms emerge as a potential influencing factor [10]. The genetic influence was observed with caffeine supplementation in which cyclists with GG genotype for CYP1A2 (C/A) polymorphism in cytochrome P450 displayed a greater ergogenic effect compared to the C allele [11]. When pequi oil was given to runners, TG genotyped athletes showed an aerobic advantage compared to GG genotyped athletes [12]. In addition, soccer players with CC genotype for the C34T polymorphism of the AMPD1 gene had a better response to creatine supplementation [13].

Since previous studies show that the increase in physical performance provided by grape juice was accompanied by antioxidant activity, we can assume that one of the genes that controls antioxidant activity, superoxide dismutase 3 (SOD3), can modulate the response of this enzyme and explain the better performance promoted by grape juice. In fact, the SOD3 enzyme is the main plasma SOD and is released by the interstitial space which participates in the elimination of superoxide (O2-) [14]. Interestingly, a naturally occurring Arg213Gly polymorphism in this binding region [15] increases the extracellular SOD concentration in the plasma of homozygous individuals 10- to 30- fold [16]. Furthermore, the Peroxisome Proliferator-Activated α-Receptor (PPARα) regulates both systemic redox activity [17] and the oxidative capacity of skeletal muscles [18, 19]. In addition, it is involved in mitochondrial activity, which directly influences endurance exercises [20].

Based on this evidence, we tested the hypothesis that the SOD3 Arg213Gly and PPARα 7G/C polymorphisms influence the redox balance and physical performance resulting from the supplementation with a single dose of grape juice. The goal of this study was to investigate the effect of a single dose of grape juice on performance in a time-to-exhaustion test in runners and verify the influence of SOD3 Arg213Gly and PPARα 7G/C polymorphisms on the time-to-exhaustion and redox balance in recreational runners.

## Materials and methods

### Study type and characterization of subjects

This is a randomized, double-blind, cross-over, controlled clinical trial with number RBR-4d9dmqz in the Brazilian Clinical Trials Registry (ReBEC). The sample size was established a priori, according to an increase in time-to-exhaustion from 59.2 ± 27.8 minutes to 68.4 ± 29.2 minutes in response to purple grape juice by Toscano et al. [9]. This increase represented an effect size of d = 0.92, so that a minimum of 13 subjects were assigned considering an α error of 0.05 and a β error of 0.80 using GPower 3.1 software program (Franz Faul, Universitat Kiel, Germany). However, as the subjects in this study were categorized by genotype, a higher number of participants was needed. Therefore, the study was conducted with 47 male recreational runners. After genotyping, the group with the smallest sample size (CC of the SOD3 gene) was left with 12 subjects.

**Fig. 1:**
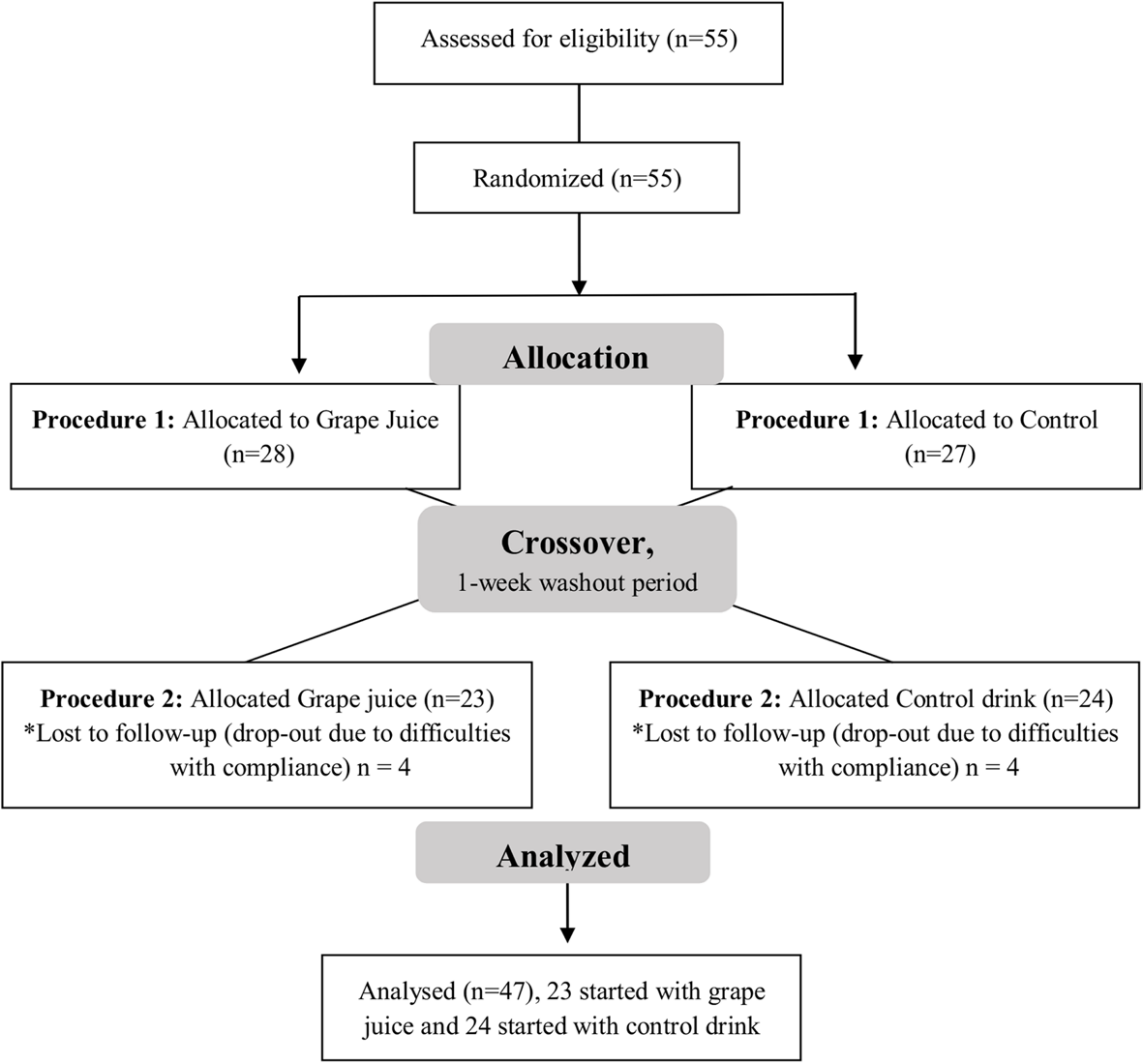
CONSORT flow diagram

Inclusion criteria were defined as recreational runners they had been training for at least one year with a frequency of at least four weekly sessions and for at least two months without interruption and participated in competitions from 5 kilometers to 21 kilometers or marathon regularly. They also not had any chronic condition or degenerative diseases, non-smokers and not using any long-term medication. Furthermore, subjects not consume purple grapes or their derivatives often; and/or dietary supplements, vitamins or bioactive substances present in the grape (polyphenols). Athletes who suffered from skeletal muscle injuries, altered dietary habits or had inconsistent training patterns, started using medication or did not perform some of the study experimental procedures were also excluded. Figure 1 shows the randomization, allocation and follow-up of subjects, according to the inclusion criteria.

### Study design

**Fig. 2:**
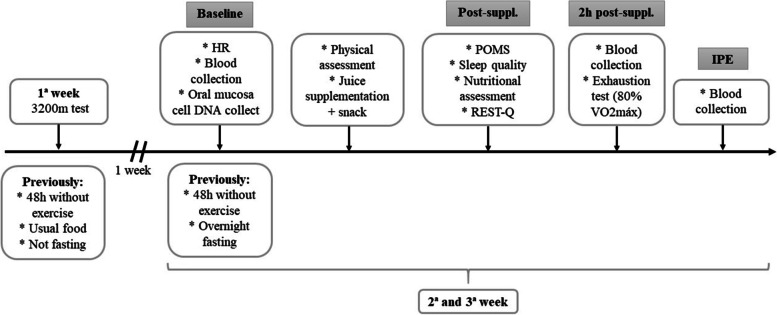
Experimental design. HR = Heart Rate, POMS = profile of mood states, RESTQ = sport recovery-stress questionnaire for athletes, VO_2_max = maximum oxygen volume, Post-suppl. = Post-supplementation, 2h post-suppl. = 2 hours post-supplementation; IPE = immediately post-exercise

Figure 2 shows the experimental design of the study. First, the athletes performed the maximum aerobic capacity test. The next 2 weeks they participated in the experimental procedures by performing the test run to exhaustion, drinking grape juice or control drink two hours prior to the test. The order of these procedures was determined randomly (in blocks) by a researcher who did not participate in the other experimental protocols using www.randomizer.org. Subjects (athletes) and the researcher, who gave the test to exhaustion, were blinded as to the supplementation tested. Heart rate and perception of effort were recorded every 10 minutes throughout the test. Blood samples were taken before supplementation (grape juice or control drink), immediately before the running test until exhaustion (two hours after supplementation) and immediately at the end of the test to measure the redox balance, muscle damage and plasmatic nitrite. Furthermore, quality of sleep, social stress and self-referred recovery were evaluated before each test. An oral mucosa sample was collected at the end of the experiment for DNA genotyping.

### Aerobic capacity test

Volunteers performed a 3200-m running test in the week before implementing the experimental protocol in order to characterize their aerobic capacity according to the protocols proposed by Weltman et al. [21]. Running tests to estimate maximum aerobic capacity were carried out with each athlete individually. The adopted 3200-m test protocol was performed on a 400-m lane of an official athletic track certified for international competitions. The athletes performed a 5-minute warm-up on the treadmill with spontaneous speed. They were instructed to start the test and complete the proposed distance in the shortest time possible. VO2max was estimated using the following equation: VO2max (ml.kg-1.min-1) = 118.4 – 4.774 x (T), where T is the time in minutes and decimal fraction.

### Pre-experimental procedures

The athletes did not exercise for 48 hours and remained 10 hours overnight fasting prior to testing and were instructed to not consume grapes or derivatives, nor eat any foods rich in antioxidants. Moreover, they did not consume caffeine or alcoholic drinks in the 24 hours prior to the test sessions.

The volunteers arrived at the laboratory at 6:30 am to start the tests on the two days of experimental protocols. First, they were acclimatized in an air-conditioned room at 22 and 25 °C, RH 65% and sat at rest for 10 minutes before starting the experimental protocol.

### Body composition and nutritional assessment

The athletes were characterized for body composition by measuring height with a portable stadiometer (Sanny, Standard, São Paulo, Brazil), and then body composition was assessed using Bioimpedance (InBody 570 Biospace®, San Francisco, California, USA), with multifrequency analysis on an eight-point tetrapolar electrode system. They were also instructed not to use caffeinated or alcoholic beverages during the previous 24 hours and not to drink water on the morning of the evaluation.

Food intake was evaluated using a 24-hour food record [22] employed twice before the time-to-exhaustion tests. This was done to characterize food consumption in the 24 hours prior to the experimental tests (Avanutri®, Rio de Janeiro, Brazil).

### Characterization of grape juice and supplementation protocol

The experimental beverage employed in this study was purple grape juice from Cooperativa Vinícola Garibaldi (Garibaldi, Serra Gaúcha, Brazil) produced from grapes of the Isabel, Bordeaux and Concord (V. labrusca) varieties. According to the manufacturer, the juice was characterized as being a natural, whole (100% grape juice) and non-alcoholic drink with no added sugar, water, flavorings or preservatives. Furthermore, 200 mL of the grape juice contains 130 kcal and 32 g of carbohydrates and does not contain a significant amount of protein, total fat, saturated fat, trans fat, dietary fiber or sodium.

The antioxidant composition was determined by free radical scavenging activities using DPPH (2,2 diphenyl-1-picrylhydrazyl) according to Brand-Williams, Cuvelier and Berset [23], and ABTS (2,2′-azinobis-3-ethylbenzthiazoline-6 sulfonic acid) [24, 25] are shown in Table 1. The total phenolic content was determined according to the methodology described by Folin-Ciocateau [26]. The quantification of specific classes of polyphenols was performed in the present study, in which the quantification of the phenolic compounds was performed by high-performance liquid chromatography (HPLC) using a Waters 2695 Alliance system (Milford, MA, USA) equipped with a diode array detector (DAD) and fluorescence detector (FLD) according to a method validated by EMBRAPA. The control drink was (maltodextrin) artificial grape flavor, which presented the same amount of carbohydrates, calories and volume [8], but without polyphenols.

**Table 1:** Antioxidant characteristics of purple grape juice

Total monomeric anthocyanins (mg/L)	ND
Trans-Resveratrol (mg/L)	0.5
Cis-Resveratrol (mg/L)	0.3
Caffeic acid (mg/L)	4.0
Caftaric acid (mg/L)	67.1
Chlorogenic acid (mg/L)	8.4
Proanthocyanidin B1 (mg/L)	2.6
Proanthocyanidin B2 (mg/L)	2,4
Epigallocatechin gallate (mg/L)	1.8
Catechin (mg/L)	1.3
Isoquercetin (mg/L)	2.3

*ND* not detected. Dates obtained for HPLC high-performance liquid chromatography, *EMBRAPA* empresa brasileira de pesquisa agropecuária

The athletes had a standardized breakfast which consisted of a sandwich (50 g of whole meal bread + 34 g of processed white light cheese = 152 kcal; 21.8 g of carbohydrates; 7.4 g of proteins; 3.6 g of fat; 4.0 g and purple grape juice or control drink, according to previous randomization. They received 10 mL/kg/day [9] of supplementation of grape juice or control drink two hours before the running test to exhaustion, so that they could reach greater bioavailability of the polyphenolic compounds present in the drink [27]. Volunteers and researchers involved in the experimental procedures were blinded to supplementation. The beverages also had similar color and flavor.

### Subjective stress, sleep and recovery/rest

To characterize the physiological conditions in the two pre-experimental test moments, the Profile of Mood States questionnaire (POMS) [28] was given to each athlete to examine the psychometric state of Total Humoral Disturbance (THD). In addition, the Stress and Recovery Questionnaire for athletes (RESTQ-Sport) [29] was used to evaluate the state of stress and recovery from everyday situations that are potentially stressful and restful. The EPWORTH-Brazilian Sleepiness Scale (ESS-BR) [30] was employed to assess the occurrence of daytime sleepiness.

### Run to exhaustion test

The athletes performed a free warm-up of between 5 and 7 minutes before starting the test. Next, they performed the running test to exhaustion on a treadmill ergometer (Moviment, São Paulo, Brazil), with the speed set at 80% of VO2max [31, 32], converting this value to belt speed according to a previous study [33]. Heart Rate (Polar FT1, Kempele, Finland) and the Borg Rating of Perceived Exertion Scale [24] was monitored every 10 minutes during the test. The test was interrupted when the athlete demonstrated inability to keep up with the treadmill speed and through verbal confirmation between 19 and 20 on the effort scale. The total running time was recorded at the end of the test. The electronic panel of the treadmill was covered so that athletes could not see the race data, especially the time elapsed in order to ensure methodological rigor. The test was performed under controlled temperature and relative humidity (RH) (22 and 25 °C, RH 65%) respectively, measured by a thermohygrometer (TFA HT-7429, São Paulo, Brazil).

### Genotyping

Samples of the oral epithelial cells in the subjects were collected by mouth washing with sucrose, and then the DNA was extracted. The genotypes were determined using the RFLP technique (Restriction Fragment Length Polymorphism) in polymerase chain using specific primers. The sense primer (5'-CGCCAGGCGCGGGAACACTCAG-3') and antisense primer (5'-GGCGGACTTGCACTCGCTCTCG-3') were used for the SOD3 Arg213Gly polymorphism. Amplification was performed according to a protocol described by Laddha [35]. The PCR product was digested using the restriction enzyme MwoI (New England 307 Biolabs, Ipswich, MA, USA) and taken to a dry incubation at 60 °C for 3 hours.

The sense (5'-ACAATCACTCCTTAAATATGGTGG-3') and antisense primers (5' -AAGTAGGGACAGACAGGACCAGTA-3') were respectively used for the PPARα 7G/C polymorphism. Amplification occurred according to the method proposed by Pishva et al. [36]. The PCR product was digested using the Taqα I enzyme (New England 316 Biolabs, Ipswich, MA, USA) and taken to incubation at 65 °C for 3 hours. Next, they were evaluated by electrophoresis on 15% polyacrylamide gel and staining with 0.5% silver nitrate.

### Redox balance, plasma nitrite and muscle damage

The oxidative activity was evaluated by lipid peroxidation that could be quantified by the metabolic product of malondialdehyde (MDA) through a thiobarbituric acid reaction in plasma [37]. The antioxidant activity was determined through total antioxidant capacity (TAC). It was quantified in plasma via the free radical scavenging activity of 2,2-diphenyl-1-picrylhydrazyl [23]. Plasma nitrite concentrations were determined by the Griess reaction that quantifies the nitrite in the sample through the diazotization reaction, forming a pinkish chromophore [38]. Muscle damage was measured by Creatine kinase (CK) using the catalytic activity method. Lactate dehydrogenase (LDH) concentrations were measured using the pyruvate-lactate method via specific commercial kits (Labtest, Minas Gerais, Brazil). The absorbances were obtained in Labmax 240 premium automatic analyzer (Labtest, Minas Gerais, Brazil) at a wavelength of 340nm.

### Statistical analysis

Data were presented as mean ± standard deviation (SD). Normality and homogeneity of data were assessed using the Shapiro-Wilk and Levene tests, respectively. Data that did not show a normal distribution were ranked according to Templeton [39] for normalization. Analyses were performed by being subdivided according to the genotype considering the presence or absence of the allele with the polymorphism characteristic. Athletes were divided based on the presence of the G allele (GG+CG genotypes) or CC genotype for the SOD3 Ag213Gly polymorphism; while subjects were categorized according to the presence of the C allele (CC+GC genotypes) or GG genotype for the PPARα 7G/C polymorphism. An independent t-test was used in order to compare the results of pre-experimental conditions (nutritional intake, sleep, mood, general stress, physical recovery and plasma biomarkers) and differences between absolute deltas in time-to-exhaustion. The results for biomarker levels were analyzed using two-way ANOVA for multiple comparisons with Bonferroni's post-hoc test. The effect size was calculated using the Cohen’s d test for independent samples using the GPower Statistics 3.1 program and then classified according to Cook, Cook and Therrin [40] into small (d = 0.00 - 0.40), medium (d = 0.50 - 0.70), large (d = 0.80 - 1.00) or very large (d ≥1.30) effect sizes. This provided information on the power of the practical application of the intervention. Moreover, an individual analysis (simple subject analysis) was performed on the exhaustion time variable to demonstrate the variability of the athletes’ responses in running performance. P-values < 0.05 were considered statistically significant. SPSS was used for data treatment (v. 23, IBM SPSS, Chicago, IL, USA).

### Ethical statement

The study was conducted entirely in accordance with the Declaration of Helsinki. Its protocol was approved by the Research Ethics Committee of the Center for Health Sciences, Federal University of Paraiba (protocol no. 2.196.523). The subjects signed an informed consent form according to Resolution 466/12 of the National Health Council (Brazil), and informed consent was also obtained from all of the subjects before the inclusion in the study.

## Results

### Characterization of experimental and control drink

The grape juice had 13.0 μMol Trolox/mL of DPPH, 9.5 ATT μMol Trolox/mL of ABTS and 3106.6 mg/L of total phenolics. The main polyphenols found in the experiments of the present study are shown in Table 1. No significant values of antioxidant compounds were found in the control drink (maltodextrin).

### Characterization of participants

Baseline characteristics of the general group and the experimental group categorized by genotypes are shown in Table 2. It was observed that the CC genotype was more frequent 35 (74.5%) in the Arg213Gly polymorphism of the SOD3 gene, followed by the CG genotype 11 (23.4%), while the GG genotype (characteristic of the polymorphism) was observed in only one athlete (2.0%). The frequency found for the PPARα 7G/C polymorphism was 28 (60.7%) for the GG genotype; 15 (32.2%) for GC and 4 (7.1%) for the CC genotype. Therefore, because there was almost an absence of GG genotype in SOD3 Arg213Gly, the group was categorized according to the presence of the allele in: (CG+GG or CC). Moreover, the categorization for PPARα 7G/C was according to the presence of the allele in: (GC+CC or GG).

**Table 2:** Baseline characteristics of athletes from the general group and the genotyped experimental group

		SOD3 Genotypes		PPARα Genotypes	
	General (*n*=47)	CG+GG (*n*=12)	CC (*n*=35)	GC+CC (*n*=24)	GG (*n*=23)
Age (years)	35.2 ± 8.6	34.5 ± 7.9	35.4 ± 8.9	35.4 ± 9.1	34.9 ± 8.2
BMI (kg.m^2^)	23.3 ± 2.7	22.6 ± 2.1	23.5 ± 2.8	22.9 ± 2.7	23.6 ± 2.7
Body fat (%)	15.1 ± 5.3	12.3 ± 6.0	16.0 ± 4.7*	14.3 ± 5.5	15.8 ± 5.1
RHR (bpm)	56.3 ± 7.6	56.0 ± 6.0	56.4 ± 8.2	57.7 ± 7.8	55.9 ± 7.4
VO_2_max (ml.kg^-1^.min^-1^)	51.9 ± 7.6	54.4 ± 8.3	51.0 ± 7.3	51.3 ± 7.7	52.5 ± 7.6
Training (years)	7.1 ± 6.4	6.5 ± 5.0	7.3 ± 6.8	6.4 ± 6.1	7.8 ± 6.7
Training frequency (days/week)	4.3 ± 1.1	4.4 ± 1.0	4.3 ± 1.1	4.1 ± 1.0	4.5 ± 1.2
Training volume (km/week)	40.5 ± 20.4	43.7 ± 15.9	39.4 ± 21.8	40.0 ± 20.1	40.4 ± 21.2
Complementary activity (min/week)	128.6 ± 96.7	116.0 ± 86	135.1 ± 101*	137 ± 92.9	120 ± 102
Work (h/day)	6.9 ± 2.3	6.9 ± 2.4	6.9 ± 2.3	7.2 ± 2.0	6.6 ± 2.5

*BMI* body mass index, *RHR* resting heart rate, *VO*_*2*_*max* maximum oxygen consumption. Data are presented as mean ± SD. (*) indicates statistical difference considering (*p* < 0.05) in the *t* test for independent samples

The runners had maximum oxygen consumption below elite runners [41], characterizing them as recreational athletes. They were young adults, with anthropometric characteristics of eutrophic and body fat characteristic for runners [42]. They had at least six and a half years of training in running and trained at least four times a week, with an average weekly distance of 40 km. In addition to running training, they performed complementary physical activities, such as cycling, swimming, team sports, wrestling, weight training and pilates. The genotypic groups were similar in all of these characteristics, except athletes with CC genotype for the SOD3 gene who performed more weekly complementary activities (*p* < 0.04) and presented a higher body fat percentage (*p* < 0.03).

Table 3 depicts that athletes in the grape juice and control drink procedures were similar at baseline conditions with the exception of plasma nitrite and CK. These were higher in the control procedure (*p* < 0.03 and *p* < 0.04, respectively). The redox balance, nitrite and muscle damage biomarkers were similar for different genotypes. The intake of macronutrients and micronutrients was similar in the experimental and control procedures. When subdivided by genotype, carbohydrate intake was higher in the group with GG+CG genotype for the SOD3 gene than in the group presenting the CC genotype (*p* < 0.00). There was a difference in zinc intake for the PPARα, which was higher in athletes with GG genotype (*p* < 0.00).

**Table 3:** Baseline variables of biochemical markers, nutritional status and physiological self-reported conditions of athletes

			Genotype SOD3		Genotype PPARα	
	Control (*n *= 47)	Grape juice (*n *= 47)	GG+CG (*n *= 12)	CC (*n *= 35)	CC+GC (*n *= 24)	GG (*n *= 23)
**Plasma biomarkers**						
TAC (%)	27.9 ± 9.3	30.4 ± 10.6	30.2 ± 10.3	30.5 ± 10.9	29.4 ± 8.4	31.5 ± 12.7
MDA (μmol/L)	3.7 ± 1.3	3.7 ± 1.3	4.0 ± 1.0	3.6 ± 1.4	3.8 ± 1.3	3.8 ± 1.3
NO (μmol/L)	11.4 ± 5.2*	9.6 ± 4.4	9.3 ± 4.9	9.7 ± 4.2	9.5 ± 4.1	9.7 ± 4.7
CK (U/L)	175.5 ± 77*	162.1 ± 55.4	155.6 ± 61.5	164.3 ± 53.9	169.8 ± 59.4	154.4 ± 51.4
LDH (U/L)	286.3 ± 44.6	293.5 ± 53.8	277.6 ± 60	299.1 ± 51.2	299.1 ± 46.9	287.9 ± 60.4
**Nutritional intake**						
Energy (kcal/kg/d)	34.3 ± 13	31.9 ± 9.6	32.6 ± 14.5	31.7 ± 7.5	30.6 ± 10.3	33.3 ± 8.8
carbohydrate (g/kg/d)	4.6 ± 2.0	4.3 ± 1.6	5.4 ± 1.8*	3.9 ± 1.4	4.6 ± 1.5	4.0 ± 1.7
Protein (g/kg/d)	1.4 ± 0.5	1.3 ± 0.5	1.3 ± 0.5	1.3 ± 0.4	1.1 ± 0.4	1.5 ± 0.4
Fat (g/kg/d)	1.1 ± 0.6	1.0 ± 0.5	1.1 ± 0.4	1.0 ± 0.5	1.0 ± 0.5	1.0 ± 0.4
Vitamin A (RE/d)	1414 ± 4734	1154 ± 2421	647 ± 2521	1313 ± 2404	1038 ± 2.699	1269 ± 2162
Vitamin C (mg/d)	110.6 ± 120	90.5 ± 101	110.3 ± 90	84.3 ± 105.6	65.3 ± 105.5	118 ± 92.1
Vitamin D (mcg/d)	7.1 ± 28.5	12.4 ± 35.8	13.3 ± 40.4	12.2 ± 34.9	11 ± 33.8	13.9 ± 38.8
Vitamin E (mg/d)	19.9 ± 21.4	14.5 ± 13.4	13.4 ± 11	14.9 ± 14.3	15.1 ± 13.6	13.9 ± 13.4
Cooper (mg/d)	1.7 ± 4.0	1.1 ± 1.1	1.2 ± 0.7	1.1 ± 1.2	1.1 ± 1.2	1.2 ± 0.9
Selenium (μcg/d)	76.2 ± 37.5	61.7 ± 43.9	66.5 ± 50.3	60.1 ± 42.2	64.9 ± 44.9	58.6 ± 43.7
Manganese (mg/d)	109.4 ± 5.1	111.6 ± 109.4	150.6 ± 105.7	99.3 ± 109.1	104.3 ± 104	118 ± 116.4
Zinc (mg/d)	8.8 ± 5.1	8.0 ± 4.0	9.0 ± 3.4	7.6 ± 4.1	6.5 ± 3.3	9.6 ± 3.8*
**Sleep, mood, general stress and physical recovery**						
Sleep (h/d)	7.1 ± 1.2	7.2 ± 1.2	7.2 ± 1.0	7.5 ± 1.2*	7.2 ± 1.4	7.3 ± 1.0
ESS-BR (score)	5.4 ± 3.2	5.5 ± 3.1	5.1 ± 3.0	7.0 ± 3.3	6.6 ± 3.2*	4.5 ± 2.7
PTH (score)	90.3 ± 15.2	90.8 ± 16.4	102 ± 21.5*	86.9 ± 12.5	92.1 ± 17	89.5 ± 18
Stress RESTQ-sport (score)	0.7 ± 0.7	0.8 ± 0.8	1.0 ± 0.9	0.6 ± 0.7	0.8 ± 0.8	0.5 ± 0.7
Recovery RESTQ-sport (escore)	3.5 ± 0.9	3.7 ± 0.9	3.5 ± 0.9	3.5 ± 0.9	3.6 ± 1.0	3.7 ± 0.8

*TAC* Total Antioxidant Capacity, *MDA* malondialdehyde, *TAC* total antioxidant capacity, *CK* creatine kinase, *LDH* lactate dehydrogenase, *ESS-BR* Epworth sleepiness scale-brazilian, *PTH* Perturbação Total de Humor, *RESTQ-sport* recovery-stress questionnaire for athletes. Data are presented as mean ± SD. (*) indicates statistical difference considering (*p* < 0.05) in the *t* test for independent samples

Physiological self-reported conditions prior to each test (sleep, mood, general stress and physical recovery) were similarly comparable when contrasting both procedures. When subdivided by genotypes, sleep quality (hours) was superior in the CC genotype (*p* < 0.01) versus the GG+CG genotype for the SOD3 gene. Sleep quality (sleepiness) was superior in the CC+GC genotype (*p* < 0.02) versus GG genotype for the PPARα gene. The mood state component was shown to be higher in the group with GG+CG genotype (*p* < 0.00) in comparison to the CC genotype. The results were similar between each genotype for the other variables.

### Physical performance

The athletes showed significantly higher performance after supplementation with the grape juice compared to the control drink (57.2 ± 17.6 versus 54.0 ± 18.9 minutes, *p* < 0.02; d = 0.17), representing an improvement of 10.1% (0.8 km of distance). The analysis of a single subject for physical performance in the running test for each athlete showed that 31 (65.9%) of the 47 runners evaluated were responsive to grape juice supplementation and presented better physical performance, while 16 (34.1%) were not responsive.

**Fig. 3:**
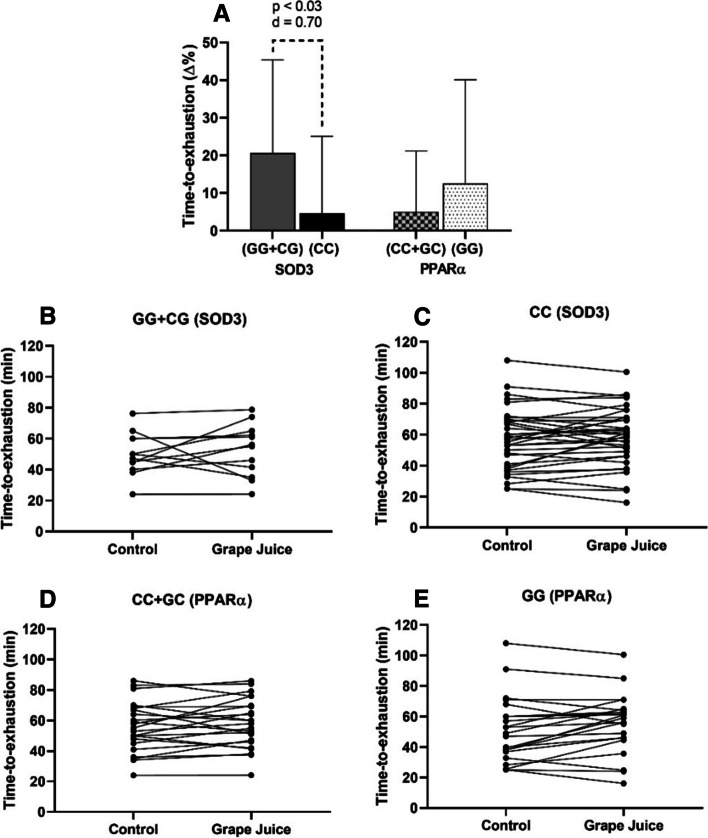
Time-to-exhaustion of recreational runners after supplementation with a single dose of whole purple grape juice, according to the genotypes for the SOD3 and PPARα genes (mean ± standard deviation). **A** relative variation from time-to-exhaustion in the genotyped groups; **B** and **C** individual analysis of the time until exhaustion, of each athlete for the SOD3 genotype; **D** and **E** - individual analysis of the time until exhaustion, of each athlete for the PPARα genotypes. *p* < 0.05 - indicates a significant difference between the groups analyzed by a paired *t* test ( *n *= 47)

The data categorized according to the genotypic groups are presented in Fig. 3 (panel A). Athletes with GG+CG genotypes showed greater time-to-exhaustion for the SOD3 gene than the CC genotype (58.4 ± 18.7 vs 53.8 ± 14.2; 3.4 ± 9.7 minutes more). This outcome was accompanied by an effect size of d = 0.70. Regarding the PPARα gene, time-to-exhaustion was similar between athletes with CC+GC genotypes (*n* = 24) and the GG genotype ( *n* = 23).

The individual analysis of the athlete’s performance test is displayed in Fig. 3, panels B and C for the SOD3 gene and panel D and E for the PPARα gene. For the SOD3 gene, it was verified that 10 (83.3%) out of 13 runners with GG+CG genotypes were responsive to grape juice supplementation and improved their time-to-exhaustion, while 3 (16.7%) were not responsive. However, only 21 (60%) of the athletes with CC genotype were responsive to grape juice and presented superior time-to-exhaustion, and 14 (40%) were not responsive, maintaining or worsening their time-to-exhaustion. For the PPARα gene, 16 (66.6%) athletes with the C allele were responsive to grape juice and increased their time-to-exhaustion, and 7 (33.4%) were not responsive. Finally, for athletes with G allele, 15 (65.2%) were responsive to grape juice and increased their running performance, while 8 (34.8%) were not responsive.

### Muscle damage

**Fig. 4:**
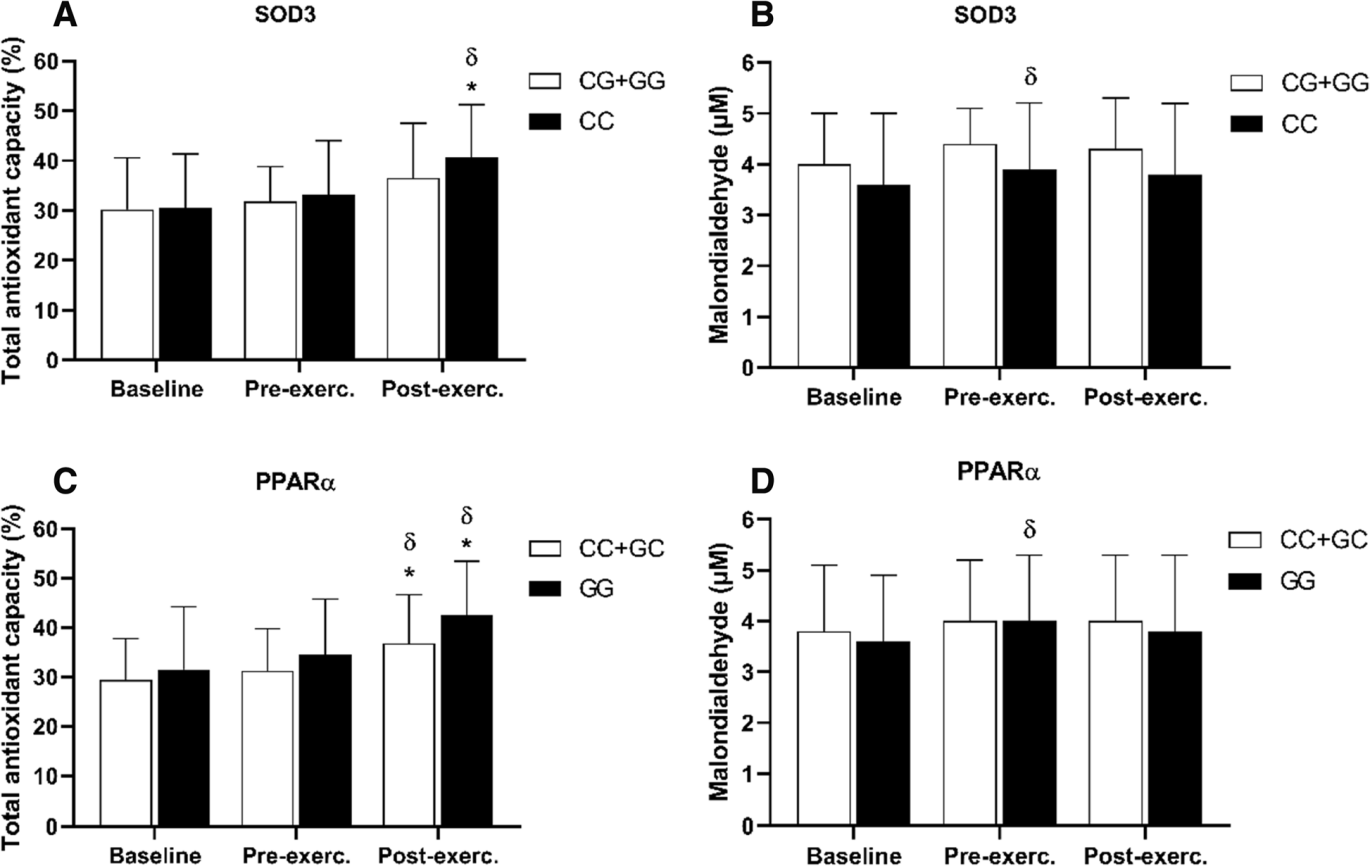
Effects of whole purple grape juice on muscle damage (mean ± standard deviation). **A** and **C** creatine kinase; **B** and **D** lactate dehydrogenase. (δ) indicates a significant difference intra-group compared with moment immediately previous; (*) indicates intra-group difference at post-exercise compared with baseline. *p* < 0.05) analyzed by a two-way ANOVA for repeated measurements (*n* = 47)

The responses of the CK and LDH enzymes in the two exercise sessions indicated that the muscle demand to the protocols were similar. Likewise, no differences were found as a function of the investigated genotypes. The SOD3 gene showed an increase in CK from pre-exercise to post-exercise in CC genotype (*p* < 0.00; d = 0.66) and GG+CG genotype (*p* < 0.00; d = 0.82). The same occurred for LDH, regarding the CC genotype (*p* < 0.00; d = 0.74) and GG+CG genotype (*p* < 0.00; d = 1.10). Both PPARα genotypes (GG: *p* < 0.00, d = 0.60; CC+GC: *p* < 0.00, d = 0.63) were associated with significant increases in muscle CK. The same occurred with LDH in the GG genotype (*p* < 0.00; d = 0.72) and finally in the CC+GG genotype in the same time (*p* < 0.00; d = 0.80) Fig. 4.

### Redox balance

**Fig. 5:**
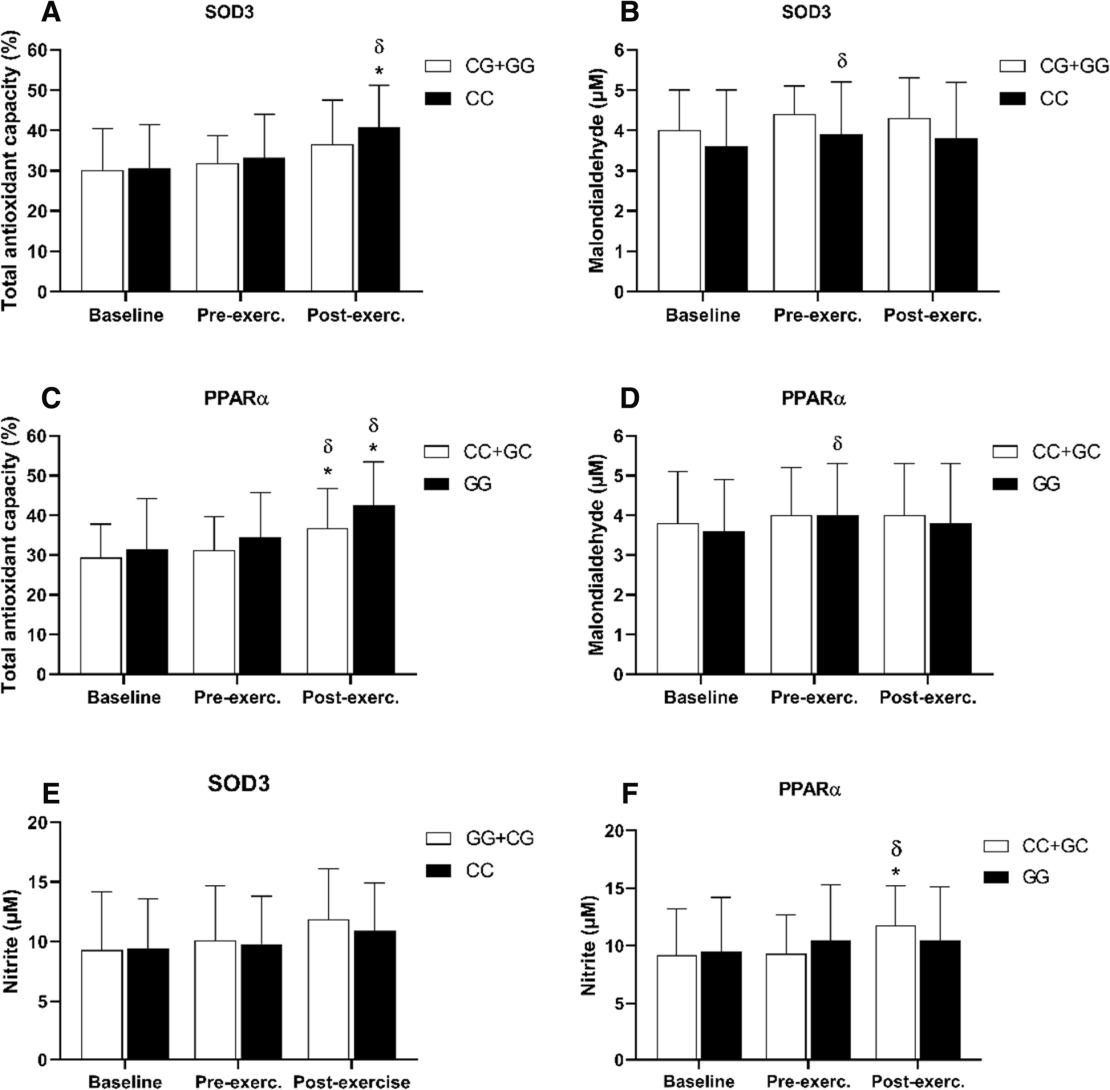
Effects of whole purple grape juice on oxidative stress (mean ± SD). **A** and **C**, antioxidant capacity; **B** and **D**, lipid peroxidation; E and F, nitrite plasmatic. (δ) indicates a significant difference intra-group compared with moment immediately previous; (*) indicates intra-group difference at post-exercise compared with baseline. (*p* < 0.05) analyzed by a two-way ANOVA for repeated measurements (*n* = 47)

The CC genotype group for the SOD3 gene (showed less improvement in physical performance) increased significantly the TAC values in the post-exercise both in relation to the baseline moment and the pre-exercise moment (*p* < 0.00; d = 1.00 and *p* < 0.00; d = 0.92), respectively (Fig. 5, panel A). However, in the group interaction analysis, the TAC values in the post-exercise moment of the CC group were not increased in relation to the GG+CG group.

In the analysis of the genotyped groups for PPARα, the TAC values increased in the post-exercise both in relation to the baseline and the pre-exercise in both groups: CC+GG (*p* < 0.00; d = 0.81 and *p* < 0.02; d = 0.62), respectively, and for the GG genotype (*p* < 0.00. d = 0.92 and 0.00; d = 0.72), respectively. However, no difference occurred between genotypes (Fig. 5, panel C).

The SOD3 or PPARα gene did not influence the responses to the malondialdehyde levels which did not undergo significant change in post-exercise. A single difference found was an increase in the baseline for pre-exercise in the CC genotype of SOD3 (*p* < 0.02; d = 0.22) and GG genotype of the PPARα gene (*p* < 0.00; d = 0.30), but without differences in the comparison between groups. In addition, the exercise had not yet been carried out at that time.

Regarding plasmatic nitrite, only the CC+GC genotype of the PPARα gene increased significantly from baseline to post-exercise (*p* < 0.01; d = 0.69) and from pre-exercise to post-exercise (*p* < 0.00; d = 0.73), but without differences in the comparison between groups.

## Discussion

This study showed that the effect of grape juice supplementation on improving performance in the run-to-exhaustion test is influenced by the GC+CG genotype of the SOD3 gene although an increase in antioxidant activity took place. This phenomenon did not explain the better performance presented by the GC+CG of the SOD3 gene group.

This study reinforces a trend of previous data reported in other research that indicate the ergogenic potential of purple grape juice to increase time-to-exhaustion in recreational runners [8] and vertical jump performance in handball athletes [43]. While the increase in performance occurred after four weeks of supplementation in these other studies, our research corroborated more recent data [9, 44] that indicated the need for only a single dose of grape juice to notice the increase in the time-to-exhaustion in recreational runners and physically active men, respectively. It also confirmed in humans the previous data found on the increase in antioxidant activity observed in studies with animal models exposed to exercise [5, 6, 45].

A wide variation in responsivity and magnitude of improvement shown to be responsive in previous studies [8, 9] were also both noted in the present study. Approximately 30% of the athletes in other studies did not respond to grape juice ergogenic supplementation when compared to a placebo or control drink. This was also the case for the data from the present study.

Our data indicated that Arg213Gly polymorphism of the SOD3 gene was a significant influence on the effect of grape juice on running performance. These results highlighted that the substitution of arginine for glycine redistributed the matrix SOD3 enzyme to extracellular fluids [46], increasing the levels of the antioxidant enzyme superoxide dismutase [47]. In fact, the beneficial effect of antioxidant supplementation on the redox balance is dependent on endogenous adaptation [48], such as the regulation of signaling pathways for mitochondrial biogenesis in response to exercise [49, 50].

Interestingly, the CG+GG group of the SOD3 gene had a significantly lower percentage of fat than the CC group. This could be an influencing factor in the performance test result. To solve this issue, we performed a correction in the test between the fat percentage and the performance delta, but we did not find any correlation between these variables (R = -0.05; *p* < 0.73).

The data demonstrated that the antioxidant response promoted by purple grape juice was not influenced by any of the genotypes studied. In fact, TAC was elevated in the post-exercise moment only in athletes with the CC genotype, and not in the GG+CG genotype that improved physical performance. However, the absence of differences between groups makes this data less consistent. Given this, we assumed that the redox balance was not a way to explain the best responsiveness of the GG+CG genotype of the SOD3 gene to grape juice in physical performance. Thus, the mechanisms explained this better responsiveness still needs to be further explored.

Despite these possibilities, our data showed that the markedly superior performance observed in subjects with the GG+CG genotype of SOD3 gene was not accompanied by greater total antioxidant capacity or lower lipid peroxidation. A possible explanation for this paradoxical outcome is that the antioxidant action data mediated by SOD3 enzyme was not demonstrated in resting conditions. Therefore, this data cannot be directly attributed to exercise.

Another possible explanation regarding this is in a methodological limitation because since the protocol was performed with humans, it was not possible to evaluate the redox balance conditions in muscle cells. As a result, the TAC and MDA plasma concentrations may not reflect muscle activity during exercise when representing the activity of the whole organism. Finally, it would be necessary to explore more direct biomarkers, such as superoxide dismutase, catalase, protein carbonylation and glutathione peroxidase.

Another way to research this includes the hypothetical possibility that the substances in grape juice could act directly on the aerobic and anaerobic energy production systems during exercise. This could contribute in some way to minimize muscle acidosis for the same exercise intensity.

In fact, oxidative stress is involved in muscle fatigue because it impairs muscle contraction mechanism, thus, contributing to the loss of physical performance during strenuous exercises [51]. However, further studies are needed to confirm this assumption, or even to determine other mechanisms involved in delayed fatigue pathways correlated to the effect of polyphenols, including the hypothesis of influence on muscle acidosis. In vitro protocols with a technique for measuring the reactivity of muscle samples in electrically stimulated organ bath conditions are an alternative to test these possibilities.

In addition to the need for future studies to confirm the possibility of the influence of oxidative stress on fatigue. It is likely that other factors beyond antioxidant activity explain the beneficial effects of grapes, such as anti-inflammatory [4, 52], cardioprotective [53, 54] and neuroprotective [55]. However, these mechanisms are poorly investigated to explain the ergogenic effect of grapes. Therefore, they deserve to be better investigated, and maybe, help to elucidate this question.

Regarding the PPARα gene results, the 7G/C polymorphism had no influence on the study variables. Our hypothesis was that the presence of the CC genotype would result in sport disadvantage because its phenotype implies an inferior response to the regulation of lipid metabolism, mitochondrial activity, biogenesis, antioxidant and anti-inflammatory defense [56]. Taken together, these phenotypes contributed to lower performance in exercise training. This was the goal of our research study. In addition, the use of a single dose of grape juice may not be enough to demonstrate an association with these phenotypes, such as improved redox balance and decreased muscle damage.

Although the present work does not confirm the association of this polymorphism with time-to-exhaustion in running, other research groups have demonstrated the influence of this polymorphism in sports performance. The frequency of the GG genotype for the PPARα gene in rowers, endurance athletes and soccer players is high [20]. This genotype is linked to improved performance by conserving glycogen reserves and using the oxidative pathway of fatty acids more efficiently. It is ideal for endurance activities [57, 58]. However, these studies only examined polymorphism without considering nutrigenetics.

The data from our study, in particular with regard to the Arg213Gly polymorphism of the SOD3 gene, brings a new element to the current basis of sports nutrigenetics. Although some foods, nutraceuticals and supplements have functional or ergogenic properties, considerable individual variability is always present. There are individuals who do not respond to dietary interventions effectively [59].

Meanwhile, previous studies have already demonstrated the influence of other genes. Womack et al. [11] found that AA homozygotes of the CYP1A2 gene had better physical performance when compared to carriers of the C allele when supplemented with caffeine. Ribeiro et al. [12] observed that runners supplemented with pequi oil showed an aerobic advantage for the TG genotype EPO T/G polymorphism. Lifanov et al. [60] demonstrated that football players supplemented creatine exhibited aerobic advantage and less lactate accumulation for the CT genotype of the AMPD1 gene. Lifanov et al. [13] supplemented runners with glutathione and observed better responses to aerobic capacity for the Pro allele in Pro198Leu polymorphism of the GPX1 gene. Given this, our study is the first to present Arg213Gly polymorphism of the SOD3 gene involved in the effect of nutritional treatments on the sports performance of running athletes.

Therefore, for practical purposes, genetic evaluation emerges in clinical practice as an additional tool for individualized prescription to optimize specific sports performance. Nevertheless, further studies are necessary to create a body of evidence in the field of sports nutrigenetics with the goal to implement them in the near future. Finally, implementing nutritional strategies based on a Athlete DNA can generate a competitive advantage. This reinforces the growth of nutrigenetics as the foundation that can help athletes improve their sports potential through dietary strategies aligned with their genetic characteristics.

## Conclusions

In summary, we concluded that this study sheds new light on the following findings. First, it reinforced the results of recent studies, indicating that purple grape juice as a new food with ergogenic capacity. Second, it also reinforced the issue of individual variability in responsiveness to nutrients for sporting purposes. Finally, this study adds new outcomes to the literature, since it has identified a genetic polymorphism of the SOD3 gene as, at least, one possible genetic variant that explains the individual variability in responsiveness to purple grape juice.

## Data Availability

The datasets used and/or analyzed during the current study are available from the corresponding author on reasonable request.
